# Glucagon-Like Peptide 1 and Taste Perception: From Molecular Mechanisms to Potential Clinical Implications

**DOI:** 10.3390/ijms22020902

**Published:** 2021-01-18

**Authors:** Mojca Jensterle, Manfredi Rizzo, Andrej Janez

**Affiliations:** 1 Diabetes and Metabolic Diseases, Division of Internal Medicine, Department of Endocrinology, University Medical Centre Ljubljana, Zaloška Cesta 7, 1000 Ljubljana, Slovenia; mojcajensterle@yahoo.com; 2 Department of Internal Medicine, Faculty of Medicine, University of Ljubljana, Zaloška Cesta 7, 1000 Ljubljana, Slovenia; 3 Division of Endocrinology, Diabetes and Metabolism, Department of Medicine, University of South Carolina, Columbia, SC 29208, USA; manfredi.rizzo@unipa.it; 4 Department of Health Promotion, Mother and Child Care, Internal Medicine and Medical Specialties, University of Palermo, 90133 Palermo, Italy

**Keywords:** GLP-1, taste, tongue, obesity, sweet

## Abstract

Preclinical studies provided some important insights into the action of glucagon-like peptide 1 (GLP-1) in taste perception. This review examines the literature to uncover some molecular mechanisms and connections between GLP-1 and the gustatory coding. Local GLP-1 production in the taste bud cells, the expression of GLP-1 receptor on the adjacent nerves, a functional continuum in the perception of sweet chemicals from the gut to the tongue and an identification of GLP-1 induced signaling pathways in peripheral and central gustatory coding all strongly suggest that GLP-1 is involved in the taste perception, especially sweet. However, the impact of GLP-1 based therapies on gustatory coding in humans remains largely unaddressed. Based on the molecular background we encourage further exploration of the tongue as a new treatment target for GLP-1 receptor agonists in clinical studies. Given that pharmacological manipulation of gustatory coding may represent a new potential strategy against obesity and diabetes, the topic is of utmost clinical relevance.

## 1. Introduction

Hedonic feelings are parts of the paramount drive in mammalian physiological systems [1]. Food is certainly important in fulfilling this need. Palatable foods that make us “feeling good” contain calorie-dense sweet and fat substances [2,3]. In environments where such nutrients are readily available with little or no effort, we are not able to successfully resist these dietary challenges [4,5]. Obesity and diabetes are persistently accelerating [6], with almost half of United States’ (US) adults predicted to have obesity and about 34.4 million predicted to have diabetes by 2030 [7]. They both represent global public health burdens due to their epidemic occurrence and their association with adverse consequences. The current strategies are obviously not able to counter the complex mechanisms underlying these global epidemics. Considering that obesity is characterized as a condition of food intake above the physiological needs of the body, we should make further efforts in understanding how feeding behavior in obesity becomes dysfunctional and how to reverse it.

The gustatory system represents the first control port of quantitative and qualitative characteristics of food that provides information about substances one considers to ingest [8,9]. It analyzes chemosensoric, orosensoric, and rewarding features of food [10]. The taste perception is clearly related to food preference and food choice [4,5,11,12,13], but current evidence about a role of taste perception in the development and persistence of obesity is conflicting [14]. Alterations in metabolic status of individual can significantly affect taste perception and the choice of food [11,12,13]. Overweight or obese subjects often have a diminished perception of sweet that may result in an increase ingestion of sweet-tasting agents to fulfill their need for sweet [15]. Moreover, an elevated desire for sweet nutrients has been reported in people that are inclined to obesity [16]. Psychophysical studies provided mixed evidence comparing obese and lean subjects, finding either increased or decreased or no changes in taste perception [14].

As opposed to sight and hearing, coding of the taste is not a simple linear sensory signal that can be easily associated with perceptual experience. Complexity of gustatory coding requires progress in understanding and continues exploration [17,18]. The role of glucagon like peptide 1 (GLP-1) in the gustatory coding deserves specific attention since GLP-1 receptor agonists (GLP 1-RAs) that are well established as antidiabetic and antiobesity therapies might have a potential to modulate the taste perception.

GLP-1 is a hormone with many functions much beyond its classical role as an incretin [19,20,21]. It has been discovered in pancreas, gut, brain regions, kidney, liver, muscles, heart, and bone [21]. It reduces food intake by centrally mediated suppression of appetite and to less extent via delay in gastric emptying [21]. The effects of GLP-1 on the food choice mediated via the gustatory system has been largely unaddressed by clinical research [22].

GLP-1 activates GLP-1 receptor (GLP-1 R). The latest extensive validation of GLP-1 R using the most specific and highly sensitive monoclonal antibody proposed a novel distribution of GLP-1 R in non-human primate and human tissues [23]. The presence of GLP-1 R was confirmed in pancreas, lung, heart, kidney and in doudenum. Lower expression of GLP-1 R was confirmed in stomach and in myenteric plexus neurons of the gut. No expression occurred in the liver and the thyroid [23]. Surprisingly, the tongue has not been explored for the presence of GLP-1 R, neither in human nor in primate species [23]. Moreover, it seems that until nowadays, the tongue remains unaddressed as a potential target of GLP 1-RAs [21].

However, preclinical research provided some important evidence of the role of GLP-1 in the endocrinology of the tongue [2,24,25], that is not yet covered in the classical textbooks of endocrinology. It was demonstrated that GLP-1 is synthesized locally in taste bud cells, that GLP-1 R is expressed in the tongue, and that there exists functional continuum in sweet sensing from the gut to the tongue.

To uncover some insights into the relationship between GLP-1 and taste perception from molecular mechanisms to potential clinical implications, we decided to provide a narrative review of this topic.

## 2. Search Methods and Results

### 2.1. Data Sources and Searches

We did a systemic search of PubMed database limited to English language without limits on year of publication.

### 2.2. Study Selection

A search algorithm included terms glucagon-like peptide-1, glucagon-like peptide-1 receptor, gustatory signaling, gustatory coding, gustatory system, taste, taste sensitivity, taste perception, sweet taste, tongue, taste buds, taste bud cells, taste bud morphology, sweet taste receptors, gustometry.

### 2.3. Data Extraction

We screened all abstracts. Full articles were assessed if the information provided by abstract suggested that the article is relevant for the topic. Data extraction was conducted by all authors.

## 3. Discussion

### 3.1. Evolutionary Perspective

The viewpoint from evolutionary endocrinology is useful to elucidate non-canonical roles of GLP-1. Most commonly, the evolution of ligand and its receptor either runs simultaneously or the ligands arose before their receptors. However, the phylogeny for the glucagone like hormones and their receptors seems to be different [26]. GLP-1 R arose first from glucagon receptor gene before the divergence of fish and mammals and then the fish has lost the gene for a receptor orthologous to the mammalian GLP-1 R [27,28]. In fish, GLP-1 acts directly on the liver causing the release of glucose into the blood like glucagon. Later, GLP-1 acquired its incretin role only in mammals. This aspect indicates that tight interactions between a ligand and receptor could evolve by recruitment of a ligand, previously constrained for a different action into a new functional complex along the evolutionary timeline. The functional complex of GLP-1/GLP-1 R within the taste bud might have evolved into its role only after sweet taste took the most privileged position in the evolution of a discriminative gustatory system in primates [26].

### 3.2. Gustatory Coding

To comprehend how GLP-1 regulates gustatory coding, it is necessary to understand the physiology that underlies taste perception. Many of the cellular and molecular mechanisms involved in taste recognition, have been elucidated in the last two decades. Presently two major hypotheses describe on how taste information is processed [17]. In simplified models, the peripheral taste system uses a segregated “labeled line” feed forward coding scheme to proceed sensory information at least for the five perceptually distinct taste modalities. By contrast, gustatory processing within the central nervous system (CNS) uses “ensemble coding system” containing a broadly distributed multisensory, feed-forward and backward combinatorial pattern across many populations of neurons, including a plastic network that includes reward [10].

### 3.3. Gustatory Coding at the Periphery

#### 3.3.1. Recognition of Taste Stimuli by Taste Bud Cells

Gustatory processing initiates in the tongue, where the chemoperception of taste is triggered by nutrients after they come in contact with the receptors on the taste bud cells (TBCs) [2,10,29]. Similarity in the anatomy of the taste buds across virtually all vertebrate animals implies that taste bud anatomy may be central to their functioning [30]. Distinct receptors are involved in recognition of different taste qualities including sweet, bitter, sour, salty, and umami. A substantial line of evidence also supports the existence of a specific receptors for orosensory perception of fat [18,31].

The TBCs from heterogenous cell populations [32] form onion shaped taste buds (TBs) distributed among different papillae that are located in the tongue, soft palate, larynx, pharynx, and epiglottis [10,30]. Taste buds contain about 100 TBCs that protrude perpendicular to the surface in a parallel arrangement. Their apical side is oriented toward the taste pore, where they contact with chemicals in the month [30,33]. They have been traditionally classified into four subtypes groups, type I-IV, by their morphological characteristics [33]. Different morphological features of TBCs correlate with their cytologic, ultrastructural and functional characteristics. All TBs contain cells of all four subtypes [2,33].

Taste bud cell type I represents about 50% of the total number of TBCs. They support the structure of TBc and are involved in several actions linked to their electrophysiological and structural properties. Amiloride-sensitive sodium channel subunit located on their surface enables a perception of low salt [34,35]. Membrane-bound enzyme adenosine triphosphate (ATP)-ase degrades ATP released from the neighboring TBCs. Structurally, TBC type I possess extensive lamellar processes that wrap around the other cell types within the TB, which probably function to control the dissipation of signaling throughout the TB and isolate fluctuations of the signals to specific areas of the TB [36,37].

**Figure 1:**
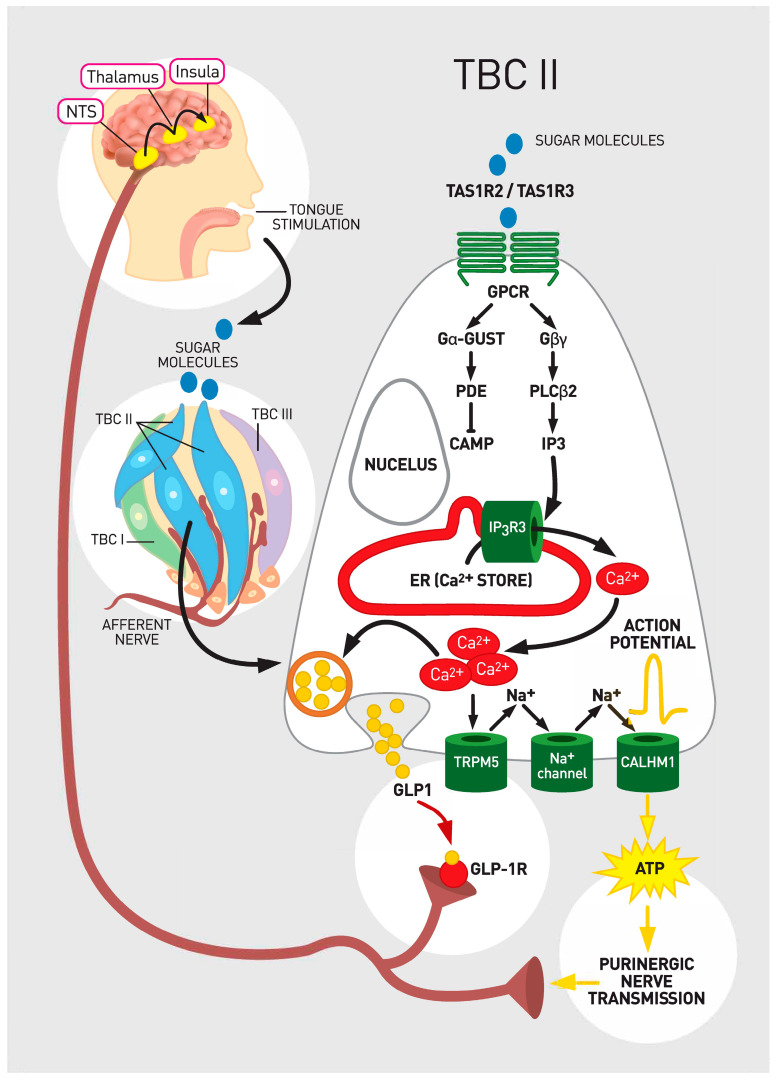
Taste sensation signaling and glucagon-like peptide 1 (GLP-1). Legend: A sugar molecule binds to heterodimeric G protein coupled receptor (GPCR) that consists of taste receptor type 1 member 2 (TAS1R2) and taste receptor type 1 member 3 (TAS1R3). Downstream signaling ultimately leads to release of ATP. Specifically, upon PLCβ2 activation IP3 as a second messenger is generated. IP3 releases intracellular Ca^2+^. Released Ca^2+^ gates transient receptor potential cation channel subfamily M member 5 (TRPM5), which results in cellular depolarization. The generated action potentials cause a release of ATP through voltage-gated calcium homeostasis modulator 1 (CALMH1) that engages purinergic receptors for ATP on the sensory nerve fibers. Sensory nerve fibers convey information to higher order neurons in the Nucleus Tractus Solitarus (NTS). Adenosine triphosphate (ATP) as a transmitter represents the major line of communication from TBC type II cells to the brain. In addition, when stimulated with sweet molecules, glucagon like peptide -1 (GLP-1) is also immediately released from TBCs by vesicular mechanisms. GLP-1 activates GLP-1 receptor (GLP-1 R) on the adjacent gustatory nerves. It seems that GLP-1 acts as ancillary neurotransmitter in cooperation with ATP for maximal activation of nerve fibers that transmit gustatory code for the perception of sweet.

Taste bud cells type II are characterized as receptor cells of the TBs. They express specific receptors and are narrowly tuned to recognition of sweet, umami and bitter stimuli [2,29]. Sweet and umami tastants are detected by heterodimeric G protein coupled receptors (GPCR). Heterodimeric G protein coupled receptors comprise of a family of three receptors from type 1 taste receptor family (TAS1R) including taste receptor type 1 member 1 (TAS1R1), taste receptor type 1 member 2 (TAS1R2), and taste receptor type 1 member 3 (TAS1R3). These proteins combine to form heterodimers that serve as the functional receptors. Heterodimeric receptors TAS1R1/TAS1R3 detect umami tastants [38,39,40,41]. Heterodimeric receptors of TAS1R2/TAS1R3 are activated by sweet chemicals [39,40,41,42,43]. Diverse parts of TAS1R2/TAS1R3 subunits enable sensation of numerous sweet molecules including glucose, fructose, galactose, sucrose, lactose, maltose, glycine, D-trypotophan, some sweet proteins such as monellin and thaumatin and artificial sweeteners [41,44,45]. The TAS1R3 subunit which detects both umami and sweet chemicals responds also to cyclamate, aspartame and neotame [44]. The TAS1R3 subunit is co-localized with alpha gustucin, one of the important components in the signaling cascade [32]. The activation of TAS1R2/TAS1R3 complex results in changes of intracellular Ca^2+^ levels [41,46]. Another cluster of GPCRs comprises of type 2 taste receptor (TAS2R) family with around 30 members. They sense bitter chemicals [47,48,49]. Each TBCs type II cell expresses specific receptors of either TAS1R or TAS2R families and responds exclusively to either sweet and umami or bitter nutrients (Figure 1). As opposed to sensation of low salt concentrations in type I cells, high salt is also sensed by type II cells [50,51].

Taste bud cells type III form neuronal synapses with sensory afferent nerve fibers in their close proximity. They are characterized as presynaptic cells. Like neurons, TBCs type III contain voltage-gated Ca^2+^ channels and release neurotransmitters including vesicular serotonin, acetylcholine, norepinephrine, and γ-aminobutyric acid (GABA) after depolarization [52]. The majority of type III cells are serotonergic [24]. In addition to their function in neurotransmission, TBCs type III are also involved in the sensation of sour (acid) [50].

The average lifespan of TBCs type I-III is approximately 10–16 days [53] and whenever they undergo apoptosis they are replenished from progenitor cells. The progenitor cells are characterized as taste TBCs type IV at the base of the TBs [54,55]. They are non-polarized, undifferentiated cells that were initially thought to be the exclusive progenitor cells [56] However, it is no longer thought that the TBC stem cell niche is located solely at the base of the TBs [53,57,58]. Sonic hedgehog protein (SHH) that regulates the differentiation of TBCs is expressed also in some cells within TBs and even outside the TBs. [53,57,58]. Accordingly, the term type IV cell is no longer uniformly used to describe a particular type of TBCs [2].

Taken together, the basic tastes may be discriminated with the receptor or electrophysiological responses by distinct TBCs subpopulations dedicated to the perceptual taste qualities [59,60,61]. The exclusive distribution of the TAS1R and TAS2R families on TBCs at the most primary stage of stimulus processing create specific coding channels regarding information about ligands that are calorically nutritive (sweet and umami), ligands that are potentially toxic (bitter) and salty or sour.

#### 3.3.2. Transduction of the Taste Signal within TBCs

Nutrient chemicals bind to the receptors on TBCs type II. The activation of the receptors triggers downstream signaling from GPCRs to a phospholipase (PLC-beta2) and a melastatin type-5 transient receptor potential cation channel (TRPM5) that is activated by a 1,4,5-trisphosphate (IP3). The induced cascade leads to increase in intracellular calcium [10].

Sweet chemicals sensed by GPCRs of TAS1R2/TAS1R3 by TBCs type II activate gustducin (the taste G- protein) dissociation into subunits [46]. The activated subunits of gustducin such as Gα and Gβγ activate phospholipase C β2 (Gβγ) resulting in generation of IP3 and diacyglyerol (DAG). IP3 causes Ca^2+^ release from the endoplasmic reticulum. Ca^2+^ opens the TRPM5 to Na^+^ influx. Increases in intracellular Na^+^ and Ca^2+^ end in cellular depolarization, generation of action potential and secretion of ATP. Subsequently the released ATP stimulates afferent neurons in close proximity to TBs [59,62,63,64] (Figure 1). The ATP that is released from TBCs type II is degraded by membrane-bound enzyme ATPases on TBCs type I, which generates ADP and prevents desensitization of ATP receptors on afferent fibers [10].

#### 3.3.3. Transduction of the Taste Signal from the TBCs to Cranial Nerves

Several classes of narrowly tuned afferent fibers of intermediary neurons, chorda tympani (CT) and glossopharyngeal (GL) nerve branching below TBs have been identified as a part of a peripheral gustatory system [9]. ATP was recognized as a key neurotransmitter and its purinergic receptor on the afferent fibers as a crucial component in taste coding at the periphery [64] (Figure 1). ATP is required to transfer information about sweet, bitter, and umami and likely salty and sour taste from TBCs to nerves [54,65] (Figure 1). Markedly diminished response to all tastes where observed in the absence of the ATP receptors on the afferent nerve fibers in genetically modified animal models [54].

Given that TBCs type II are not presynaptic cells and that TBCs type III as the classical presynaptic cells do not release ATP [66,67] it remains challenging to understand how the specificity of initial taste coding could be maintained across synapse. Additional mechanisms including some ancillary neurotransmitters are likely involved to preserve the accurate transmission of taste code from TBCs to nerve fibers [64].

#### 3.3.4. Paracrine Signaling inside the Taste Buds

Paracrine signaling between TBCs plays an important ancillary role in further processing of gustatory code during signal transmission from TBCs to nerves [68]. Such signaling enriches the information about the quality and hedonic value of the stimulus [68].

Several peptide hormones from the gut are also identified in TBCs and are involved in the gustatory coding. Glucagon-like peptide-1, glucagon, neuropeptide Y, cholecystokinin, and vasoactive intestinal peptide were identified inside TBs. Each gut hormone and its cognate receptor were restricted to subpopulations of TBCs and associated nerve fibers [11,24,69,70,71,72,73].

### 3.4. GLP-1 in the Taste Buds

GLP-1 is expressed in the two subsets of TAS1R3-immunopositive TBCs. In TBCs type II, GLP-1 expression is restricted to around one quarter of TAS1R3 and gustucin positive cells, whereas in type III cells it is restricted to some serotonin containing type III cells [24,64,72] (Table 1).

Taste cells with locally produced GLP-1 express the enzyme prohormone convertase, that is necessary for the cleavage of proglucagon to GLP-1. GLP-1 is most likely secreted from TBCs by vesicles, although the strong evidence for such mechanism is lacking (Figure 1). The amount of GLP-1 that is released form TBCs is limited, not detected in serum, and does not cause systemic effects. Initially, it was believed that TBs do not express an enzyme DPP4 that rapidly catabolizes active GLP-1 [24]. Later, DPP-4 immunoreactive cells localized in the rat taste buds with significantly higher expression of DPP-4 mRNA in the rats with diabetes [74].

When sweet molecules had activated the receptors on TBCs, GLP-1 was immediately released from TBCs [64]. Then GLP-1 activated GLP-1 R on adjacent S types of gustatory nerve fibers and stimulated a large transient response [64] (Figure 1). Afterwards, in the microenviroment around the taste bud, blood circulation likely provided enough local DPP4 to inactivate GLP-1 from taste cells to limit signal duration [64].

A deficiency of GLP-1 R in animal models demonstrated a clearly reduced sensitivity to both calorie-containing (sucrose) and artificial (sucralose) sweeteners with no difference in comparison to wild-type mice for bitter, salty, or sour [24]. In another study GLP1 R knockout mice exhibited reduced taste sensitivity to both nutritive and artificial sweeteners, but display hypersensitivity to sour tastant [24]. These two differential responses may reflect the differential effects of GLP-1 secreted from subsets of Type II and Type III cells. GLP-1 R^--/--^ mice exhibited reduced nerve responses to sweet stimuli in the tongue [64]. Surprisingly, GLP-1 R^--/--^ mice were much more sensitive to the umami stimulus, implying that GLP-1 might impact sweet and umami taste responses in distinct ways [12,24]. The effect of sweet taste stimuli was concentration dependent; higher concentrations of sweet taste stimuli produced higher GLP-1 stimulation [64].

Sucrose, artificial sweeteners and umami stimuli elicit secretion of GLP-1 and NPY from TBCs in mouse circumvallate papillae—an effect not present in TAS1R3 null mice [75]. Moreover, studies in both humans and mice have shown that long chain fatty acids increase GLP-1 secretion from TBCs in addition to reinforcing the preference for sucrose in a GLP-1R dependent manner, probably by interacting with GPR120 [18,76].

In summary, the production of GLP-1 in taste cells, the presence of the GLP-1 R on adjunct nerve fibers and the results from knockout animal experiments strongly suggest that GLP-1 signaling acts locally in taste buds and can affect taste function [12]. It likely acts as ancillary neurotransmitter in cooperation with ATP that is required for maximal activation of sweet nerve fibers [64]. Such signaling conveys information about the perceptual quality of the nutrients from the very first place of interaction of food with the individual organism.

### 3.5. Continuum of Sweet Sensation from the Gut to the Tongue

The local production of gut hormones in the TBCs demonstrates functional similarities along alimentary canal. GLP-1 and GLP-1 R are distributed from the tongue to the gut. Such distribution enables a continuous functional analysis and regulation of ingestion, digestion, absorption, and metabolic fate of the nutritive food. From molecular perspective, the sweet sensation in the tongue shares many characteristics with the sensation of sweet in the gut epitellium. [18]. Like in the tongue, glucose activates the release of GLP-1 from the gut epitellium upon its binding to the receptor. Several molecular mechanisms in the gut have been identified for this release, the classic two being closure of ATP-sensitive potassium channels and sodium-coupled glucose uptake by sodium/glucose transporters (SGLTs) [21,77]. Key part of sweet receptor TAS1R has been identified in many gut cells including K-cells, L-cells, K/L enteroendocrine cells, brush, and X/A-like cells of the stomach [78]. Like in the TBs, TAS1Rs components of GPCRs on the gut cells act as intestinal glucose sensors that lead to glucose-stimulated GLP-1 release. TAS1R2/TAS1R3 heterodimers and α-gustducin are also present in gut K/L-cells that express both GLP-1 and gastric inhibitory polypeptide, as well as in L-cells that express GLP-1 and GLP-2 [79,80,81]. The magnitude of the role of intestinal TAS1R in glucose-stimulated GLP-1 secretion in the gut remains debatable. Mice lacking α-gustducin as a part of TAS1R on type II TBCs, had clearly reduced glucose-mediated GLP-1 release from the gut [79]. Moreover, TAS1Rs regulate expression of SGLT-1 protein that is crucial for transport of sugars from the intestinal lumen into enterocytes. Mice deficient for either α-gustducin or TAS1R3 are not able to upregulate expression of SGLT-1 in the intestine when carbohydrates entering the small gut [2,80]. Upon receptor-recognition of monosaccharides and other carbohydrates on the gut epitellium, voltage-dependent Ca^2+^ channels open and influx of Ca^2+^ triggers release of GLP-1 into the circulation by vesicular exocytosis, just as in the TBs [21,82].

### 3.6. Central Taste Coding

Previous reviews yielded extensive insights into the central neural coding of gustatory information [2,9,10,17,83]. Here we only briefly discuss the central taste coding without elaborating on all the complexities.

#### 3.6.1. Taste Coding in the Brainstem and Thalamus

Nerve fibers of the facial, glossopharyngeal and vagus cranial nerves that transmit taste information from the tongue project from their specific cranial nerve ganglia to the medulla, in the nucleus tractus solitarus (NTS) [10]. Nucleus tractus solitarus converges fibers from all three cranial nerves from the tongue with efferent and afferent autonomic fibers of the vagus from the gut and with somatosensory afferent fibers from the trigeminal nerve [10].

The rodent and primate taste system at this level differ. In rodents, NTS afferent fibers convey taste information to gustatory centers of the parabrachial nucleus (PBN) in the pons that synapse with neurons in the thalamus. At or just above the PBN, one-third of the ascending nerve fibers carrying taste perception from the tongue cross and ascend bilaterally to the thalamic taste area allowing bilateral taste representation in the brain [84]. In primates, axons from the NTS bypass the PBN and project directly to the ventral posterior medial nucleus of the thalamus.

#### 3.6.2. Taste Coding in the Primary Gustatory Cortex

In both rodents and primates, thalamic afferents terminate at the primary gustatory cortex (GC) in the anterior insula of the temporal lobe, where taste coding can further be distinguished [85]. Gustatory cortex differentiates the subtleties of salty, sweet, sour, bitter, and umami. This brain area also integrates other multisensoric modalities including thermal, mechanical, visceral, and nociceptive stimuli [85,86,87]. Individual response of GC neurons to tastants are either selective to tastens or more broadly tuned. Optical imaging in animal studies identified four distinctive spatial patterns representing sweet, bitter, salty, and sour taste modalities, but no region was clearly specific to a single modality [88,89]. Similarly, functional imaging and electrophysiology studies in humans showed distinctive spatial patterns for five taste modalities as well as overlapping broad distribution of taste-responsive neurons found throughout insular cortex with no spatial organization [85,90]. The central responses to tastants has been topographically represented with different neuroimaging studies including calcium-imaging studies of single neurons and ultra-high resolution functional magnetic resonance imaging that enable one of the highest topographical resolution at a finer scale [10,90].

Projections of gustatory neurons then extend also to the amygdala and onwards to the lateral hypothalamus and mesolimbic reward system such as nucleus accumbens and ventral tegmental area [84]. At that level hedonic value is added to the taste information [91,92]. Depending on the concentration, bitter and sour chemosensations are generally experienced as unpleasurable, whereas sweet and salty tastes are pleasurable [91,92].

#### 3.6.3. Taste Coding in the Secondary Gustatory Cortex

Central gustatory pathways further dynamically interact in feed-forward and top-down pathways that are widely distributed among several brain areas [10]. Primary GC neurons project reciprocally back to the pons and forward to the primary somatosensory cortex and to the secondary taste brain area in orbitofrontolateral cortex (OFC) [10].

The OFC is recognized as the secondary taste cortex because it has direct projections from the primary GC. It lies in close proximity to the primary olfactory piriform cortex. The neurons in this area receive convergent gustatory, olfactory as well as visual and somatosensory signals. They regulate food selection, predict reward, and imprint the reward value [93]. The response depends also on the previous experience and internal metabolic state of individuals; for instance, when food is eaten to satiety it becomes less rewarding, without changing the taste of the food itself [92]. Neurons from OFC further extend to the prefrontal cortex. Prefrontal cortex encodes the reward value and the reward’s forthcoming behavioral response at the highest level of regulation [10,94].

#### 3.6.4. GLP-1 Signaling in the Central Taste Coding

Nucleus tractus solitarus acts as an important relay station that integrates peripheral gustatory signals and transmits them further to dedicated brain areas. GLP-1 is expressed locally in the PreProGlucagon Neurons in NTS [19] (Table 1). Animal studies demonstrated that direct central administration of GLP-1 elicited a conditioned taste aversion to sweet nutrients [19], what suggests that there might be some direct impact of centrally produced GLP-1 on the gustatory coding. Furthermore, GLP-1 released from the gut cells interacts with NTS via the vagus nerve, which in turn activate neurons in NTS and might indirectly contribute to central gustatory coding [19]. Similarly, neural signals elicited by GLP-1 from the tongue may also contribute to this reflex pathway because these signals are also transmitted to NTS via the chorda tympani and GL nerves [64].

Altogether, central gustatory processing contains a multisensory, distributed, feed-forward and backward, plastic network that includes reward. The central responses depend also on individual metabolic state and previous experiences [10]. Potential direct action of GLP-1 on taste modulation in the central gustatory system via NTS has been demonstrated in animal models.

## 4. Conclusions

In summary, GLP-1 is locally produced in TRCs type II and III. GLP-1 R is expressed on adjacent taste nerve fibers in the tongue. The action of GLP-1 was identified also in central gustatory coding. Consistent with this, we provided evidence about the role of GLP-1 in gustatory coding, mostly based on preclinical studies. This characterized the tongue as a potential new target for therapeutic manipulation with GLP-1 RAs.

The field should initiate the collaboration of endocrinologists, diabetologists, ear, nose, and throat (ENT) specialists, neuroscientists, and nutritionists to further explore GLP-1 in taste perception of humans. Since consumption of calorie dense palatable foods is highly pertinent to the onset and maintenance of obesity and diabetes, the potential modulation of taste sensitivity and food preference with GLP-1 based therapies is of important clinical relevance. Exploring the potential possibilities to modulate gustatory coding by pharmacological manipulation remains one of an intriguing clinical challenge.

## Figures and Tables

**Table 1:** Summary of the GLP-1/GLP-1 R effects in the gustatory coding.

Location	Production of GLP-1	Expression of GLP-1 R	Role
TBC Type II	Yes	Yes	Peripheral Gustatory Coding for sweet and umami
TBC type III	Yes	Yes	Peripheral Gustatory Coding for sour ?
Afferent Nerves in the tongue	No	Yes	Transduction of the gustatory signal from TBCs to Nucleus Tractus Solitarus
Nucleus Tractus Solitarus	Yes (PPGN)	Yes	Central Gustatory Coding

Legend: GLP-1: Glucagon Like Peptide-1; GLP-1 R: Glucagon Like Peptide-1 Receptor; TBC: Taste Bud Cell; PPGN: PreProGlucagon Neurons.
